# SPIRIT: Structural Entropy Guided Prefix Tuning for Hierarchical Text Classification

**DOI:** 10.3390/e27020128

**Published:** 2025-01-26

**Authors:** He Zhu, Jinxiang Xia, Ruomei Liu, Bowen Deng

**Affiliations:** 1 State Key Laboratory of Software Development Environment, School of Computer Science and Engineering, Beihang University, No. 37 Xue Yuan Road, Hai Dian District, Beijing 100191, China; jinxiangxia@buaa.edu.cn (J.X.); rmliu@buaa.edu.cn (R.L.); 2 School of Advanced Technology, Xi’an Jiaotong-Liverpool University, Suzhou 215213, China; bowen.deng22@student.xjtlu.edu.cn

**Keywords:** information theory, structural entropy, representation learning

## Abstract

Hierarchical text classification (HTC) is a challenging task that requires classifiers to solve a series of multi-label subtasks considering hierarchical dependencies among labels. Recent studies have introduced prompt tuning to create closer connections between the language model (LM) and the complex label hierarchy. However, we find that the model’s attention to the prompt gradually decreases as the prompt moves from the input to the output layer, revealing the limitations of previous prompt tuning methods for HTC. Given the success of prefix tuning-based studies in natural language understanding tasks, we introduce **S**tructural entro**P**y gu**I**ded p**R**ef**I**x **T**uning (**SPIRIT**). Specifically, we extract the essential structure of the label hierarchy via structural entropy minimization and decode the abstractive structural information as the prefix to prompt all intermediate layers in the LM. Additionally, a depth-wise reparameterization strategy is developed to enhance optimization and propagate the prefix throughout the LM layers. Extensive evaluation on four popular datasets demonstrates that SPIRIT achieves a state-of-the-art performance.

## 1. Introduction

Hierarchical text classification (HTC) poses a significant challenge, as the classifier must simultaneously comprehend the semantics in the text and the hierarchical correlations inherent in the label set. A popular approach for HTC is to express the label set as a label hierarchy (intrinsically, a directed acyclic graph) and adopt a graph neural network to extract structural information using a dual-encoder framework in conjunction with the LM [1]. Furthermore, to enhance the collaboration between the text and the structure encoders, various deep learning techniques, such as semantic matching [2], information maximization [3], and contrastive learning [4,5], are introduced to HTC. To fully exploit and reuse the capabilities of pretrained language models, recent studies have proposed prompt tuning methods [6,7,8], achieving great improvements in HTC.

**Figure 1:**
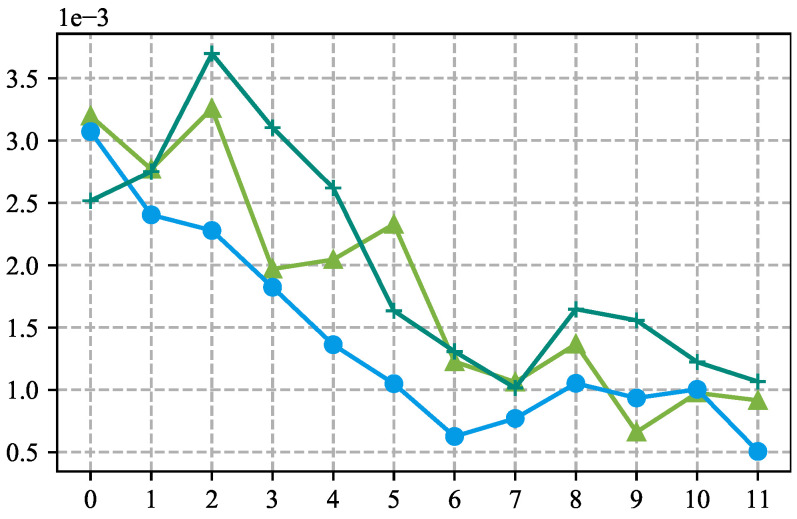
In HPT [6], the attention scores from text tokens to prompts significantly drop in the deeper LM layers. Different colored lines represent different samples.

However, two observations reveal the limitations of vanilla prompt tuning and spark our interest in prefix tuning for HTC: (1) As depicted in Figure 1, HPT [6], a prominent prompt tuning method for HTC, where the attention scores on the prompt contents potentially display continual downward trends as the input dataflows toward the output layer. (2) Other prior research indicates that prompt tuning encounters performance bottlenecks on natural language understanding tasks, whereas the involvement of prefix tuning [9,10] yields performance comparable to fine-tuning [11]. Considering the two observations, prefix tuning may be a better choice than prompt tuning for HTC, as the prefix could persistently recall the label hierarchy to the language model.

Another issue preventing the prompt learning method from breakthroughs in HTC is that natural language-based prompts fail to convey the intricate label hierarchy [6], even when using advanced language models like ChatGPT [5]. In contrast, we consider generating prompts from a structural perspective. In light of structural entropy [12], which measures the uncertainty of a graph and provides the essential structure of the graph formulated in coding trees, we aim to construct structural prefixes for HTC with the principle of structural entropy minimization. Specifically, we implement an algorithm to realize optimal coding tree construction, uncovering the underlying structure of label hierarchies. Subsequently, we design a structural prefix network to decode the structural information embedded in the coding trees and generate initial prefix representations. Additionally, a depth-wise reparameterization is originated to propagate the prompt tokens to each hidden layer, allowing prefixes to be better adapted to the LM and reduce the training burden. In comparison with other supervised-only, contrastive learning, and prompt tuning methods, SPIRIT achieves superior performance on four common datasets. Overall, the contributions of our work can be summarized as follows:

- We propose a structural entropy guided prefix tuning method named SPIRIT for hierarchical text classification. To the best of our knowledge, this paper is the first introduction of prefix tuning to HTC.
- To construct hierarchy-aware prefixes, we decode the essential structure of label hierarchies with a structural entropy minimization algorithm, providing abstractive information to be encoded into the prefix.
- Given the essential structure of label hierarchies, a structural prefix network is proposed to construct initial prefix embeddings for prompting all hidden layers in the LM. Further, a depth-wise reparameterization strategy is originated for adapting structural prefixes to the LM.
- Experiments on four datasets demonstrate the effectiveness of SPIRIT For reproducibility, source code is available at https://github.com/Rooooyy/SPIRIT (accessed on 11 January 2025).

## 2. Preliminary

**Hierarchical Text Classification.** In a specific hierarchical text classification task, there exists *L* classification subtasks $Y=\{Y^{1},Y^{2},\ldots ,Y^{L}\}$, where $Y^{l}=\left\{y_{1},y_{2},\ldots \right\}\in {\left\{0,1\right\}}^{|Y^{l}|}$ is a multi-label classification task at the *l*-th level. The total number of label is denoted as $\left|Y\right|=\sum_{l=1}^{L}\left|Y^{l}\right|$. Furthermore, dependencies among labels in adjacent levels, derived from their semantics, can be formulated as a directed graph. This graph—known as the label hierarchy—contains edges only from nodes at the (l−1)-th level to those at the *l*-th level (i.e., no self-loops or cross-level linking), capturing the hierarchical dependencies embedded in the label set. The ground truth labels always co-occur as paths from the root node to the leaf nodes. Given a piece of text $D=\{x_{1},x_{2},\ldots ,x_{N}\}$, the classifier should predict the labels in Y concerning the hierarchy.

**Structural Entropy.** Structural entropy [12] extends Shannon entropy on graph structures, measuring the uncertainty of a graph. Coding trees are carriers for structural entropy, revealing the essential structure of a graph. Formally, the structural entropy of a graph G=(V,E) is defined as follows:

(1) $$H^{T}\left(G\right)=-\sum_{\alpha \in T}\frac{g_{\alpha }}{vol(G)}\log_{2}\frac{vol(\alpha )}{vol(\alpha^{-})},$$

where α is a non-root node of coding tree T which represents a subset of $V_{G}$, $\alpha^{-}$ is the parent node of α on the coding tree. $g_{\alpha }$ represents the out degree of α on *G*. vol(G) denotes the volume of graph *G* while vol(α) and $vol(\alpha^{-})$ are the total degrees of nodes in $T_{\alpha }$ and $T_{\alpha^{-}}$, respectively. The height of the coding tree should be fixed to formulate a certain coding scheme. Accordingly, the *K*-dimensional structural entropy of the graph *G* is the minimal structural entropy determined by coding tree *T* with height no greater than *K*, formally,

(2) $$H_{K}\left(G\right)=\min_{\left\{T|height(T)\leq K\right\}}H^{T}\left(G\right).$$

**Coding Tree.** The coding tree T of a graph G=(V,E) is a tree with the following properties: (1) Every node α∈T is assigned with a non-empty subset of *V*, denoted as $T_{\alpha }$. The root node λ of T marks all nodes in *G*, i.e., $T_{\lambda }=V$. For each leaf node γ∈T, $T_{\gamma }$ contains a single node v∈G. (2) For each non-leaf node α∈T, its *i*-th child node is denoted as $\alpha^{<i>}$, $T_{\alpha }$ is the union of its child nodes, i.e., $T_{\alpha }={⋃}_{i}T_{a^{<i>}}$ (there is a proposition that nodes on the same level as T provide a partition of *V*). Additionally, a notation list is given in Appendix A.1.

## 3. Methodology

**Figure 2:**
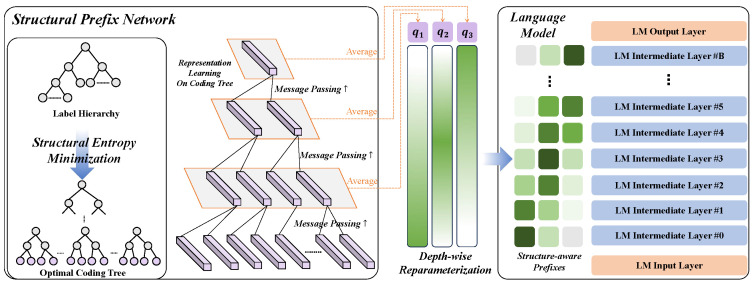
An example of SPIRIT with K=3. Given a pretrained LM, we first feed input prompt (Section 3.1) to the model, then we utilize the structural entropy guided prefix network (Section 3.2) to generate hierarchy-aware prefix tokens. The LM is fine-tuned with ZLPR loss and MLM loss (Section 3.3). After observation of the prompt and prefix tokens, the model is constrained to predict labels according to the hierarchy (Section 3.3).

In this section, we elaborate on the design of SPIRIT in Figure 2.

### 3.1. Input Prompt

Following [6], we construct dynamic soft prompts incorporating hierarchy-aware information. The quantity of virtual tokens is equal to the depth of the label hierarchy. Each token corresponds to a layer in the hierarchy with the same index. Furthermore, each layer’s prompt token is appended by a special token for layer-wise classification. Formally, given a document $D=\{x_{1},x_{2},\ldots ,x_{N}\}$, the input to the LM is as follows:

(3) $$\left\{x_{C};x_{1},x_{2},\ldots ,x_{N};t_{1},x_{P},t_{2},x_{P},\ldots ,t_{L},x_{P};x_{S}\right\},$$

where $x_{C}$ and $x_{S}$ denote the embeddings of the <CLS> and <SEP> tokens, respectively. $x_{P}$ is initialized with <MASK> token for layer-wise multi-label prediction. $\left\{t_{1},t_{2},\ldots ,t_{L}\right\}$ are prompt tokens produced by a prompt encoder. Specifically, *L* virtual nodes are added to the label hierarchy and each virtual node is linked with all label nodes in the same layer. For representation learning, the virtual nodes are randomly initialized while the label nodes are initialized with their textual description encoded by the LM. Thereafter, a one-layer graph attention network (GAT) [13] is adopted to model label correlations on the augmented label hierarchy graph. The embeddings of virtual nodes fetched by GAT are taken as continuous prompts, where the information of the label hierarchy is condensed. Given a node *u* on the label hierarchy graph within virtual nodes, the information propagation process of GAT is formulated as

(4) $$t_{u}=\mathrm{ReLU}\left(\sum_{v\in N(u)⋃u}\frac{W_{G}t_{v}}{c_{u}}\right),$$

where N(u) denotes the neighbors for node *u*, $c_{u}$ is a normalization constant, and $W_{G}\in R^{d_{G}\times d_{G}}$ is the trainable parameter. Then, we filter out those embeddings of the virtual nodes $\left\{t_{1},t_{2},\ldots ,t_{L}\right\}$ to construct the prompt.

### 3.2. Structural Entropy Guided Prefix

Simply prompting the model at input layer might not be sufficient for HTC as the label hierarchy provides rules for classification, rather than merely serving as task instructions. To continually reinforce the LM’s memory of the label hierarchy, we replay the knowledge of labels via prefix tuning [9]. This involves introducing prompt tokens for all intermediate layers in the LM. Intuitively, the intermediate layers should be more attentive to coarse-grained information compared with the input layer. Further, layers at higher levels should receive and produce denser information than those at lower levels. Based on these assumptions, we utilize the essential structural information provided by coding trees, which preserve minimal structural entropy [12], to construct prefix tokens. We also propose a depth-wise reparameterization strategy for broadcasting the prefixes to LM layers at different depths.

**Coding Tree Construction.** Structural entropy could measure the uncertainty of a graph. Minimizing structural entropy, a coding tree can be constructed to reveal the essential structure of the graph. Converting the label hierarchy into such a coding tree would make the intermediate LM layers easier to understand. To this end, we design an efficient algorithm to construct the optimal coding tree obtaining minimal *K* dimensional structural entropy. Given a graph G=(V,E), let $C=\{C_{1},\ldots ,C_{m}\}$ be a partition of *V*, where $C_{i}\subset V$ is called a community. The algorithm is implemented based on three atom functions as follows:

**Definition** **1.** *Function $F_{M}\left(\cdot \right)$. Given any two communities $C_{\alpha },C_{\beta }\in C$, merge function $F_{M}\left(\alpha ,\beta \right)$ merges $C_{\alpha }$ and $C_{\beta }$ into a new community $C_{\gamma }$, i.e., $C_{\gamma }=C_{\alpha }\cup C_{\beta }$.*

Here, we denote the reduction in structural entropy after calling $F_{M}\left(\alpha ,\beta \right)$ as $\Delta H_{K}^{\left(\alpha ,\beta \right)}\left(G\right)$. According to Equation (1), we have

(5) $$\begin{array}{cc} \Delta H_{K}^{\left(\alpha ,\beta \right)}\left(G\right)= & \frac{1}{vol(G)}\{\left[vol\left(\alpha \right)-g_{\alpha }\right]log_{2}\left[vol\left(\alpha \right)\right]\\ \; & +\left[vol\left(\beta \right)-g_{\beta }\right]log_{2}\left[vol\left(\beta \right)\right]\\ \; & -\left[vol\left(\gamma \right)-g_{\gamma }\right]log_{2}[vol\left(\gamma \right)]\\ \; & +\left[g_{\alpha }+g_{\beta }-g_{\gamma }\right]log_{2}\left[vol\left(G\right)\right]\}. \end{array}$$

**Definition** **2.** *Function $F_{S}\left(\cdot \right)$. Given a graph G and a partition C, function $F_{S}\left(C\right)$ squeezes G in the following procedure. For all communities $C_{i}$ in C, nodes marked by $C_{i}$ on graph G are squeezed to a new node $v_{i}^{+}$, and the edge weights of $v_{i}^{+}$ to others is the summation of those original nodes in $C_{i}$.*

**Definition** **3.** *Function $F_{U}\left(\cdot \right)$. Given a coding tree T and a partition C. $F_{U}\left(T,C\right)$ will update coding tree T by taking all communities in C as leaf nodes of T, leading to an increase in height.*

Initially, we adopt each node in the graph as a single community, and then iteratively execute $F_{M}\left(\cdot \right)$ and $F_{S}\left(\cdot \right)$ until a *K*-dimensional coding tree is constructed. Given a sub-graph $G_{k}$ of *G* in the *k*-th iteration, we merge the communities with the maximal $\Delta H_{K}\left(G_{k}\right)$ greedily until there are no communities satisfying $\Delta H_{K}\left(G_{k}\right)>0$, thereby achieving the minimal *K*-dimensional structural entropy. The complete procedure is shown in Algorithm 1. Furthermore, a case study is provided in Appendix A.2 for a better illustration of Algorithm 1.
**Algorithm 1: **Coding Tree Construction
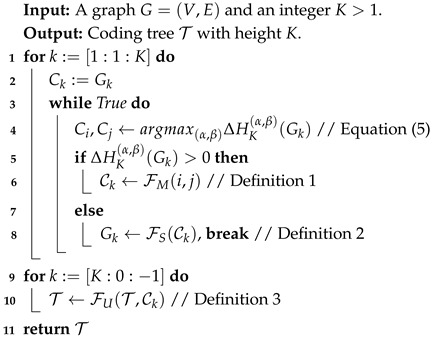

**Theorem** **1.** *Given a graph G=(V,E), for any target height K, Algorithm 1 is in the complexity of O(|V|).*

**Proof.**  Lines 1–6 of Algorithm 1 merge the nodes of T from bottom to top. For each iteration in k:=[1:1:K], $F_{M}\left(\cdot \right)$ generates $log_{2}\left(\right|C_{k-1}\left|\right)$ new nodes for $C_{k}$. Then, $F_{S}\left(\cdot \right)$ traverses and squeezes these nodes; this results in the retention of nodes no more than $log_{2}\left(\right|C_{k-1}\left|\right)$. Therefore, the node size of T is lower than that of a binary tree $T_{B}$ constructed on the |V| leaf nodes. The complexity of traversing $T_{B}$ is calculated as follows

(6) $$\begin{array}{cc} O(T_{B}) & =O(|E_{T_{B}}|)\\ \; & =O(|V_{T_{B}}|-1)\\ \; & =O(|V_{T_{B}}^{0}|+|V_{T_{B}}^{1}|+\ldots |V_{T_{B}}^{T_{B}.height}|-1)\\ \; & <O(2|V_{T_{B}}^{0}|)\\ \; & =O(2|V|)\\ \; & =O(|V|). \end{array}$$

Algorithm 1 executes *K* linear traversals and operations on T. The total number of operations is twice the node size of T. Thus, the complexity of Algorithm 1 is also O(|V|). □

**Structural Prefix Network.** After calling Algorithm 1 on the label hierarchy, we obtain a coding tree $T=\left(V,E\right),V=\{C_{k}|k\in \left[0,K\right]\}$ with height *K*. For representation learning, we initialize the leaf node embeddings $X^{0}$ with the textual descriptions of their corresponding labels. Here, non-leaf nodes embeddings $\left\{X^{k}|k\in \left[1,K\right]\right\}$ still remain unknown. To fetch intermediate node embeddings, we design a message-passing network as follows,

(7) $$\begin{array}{c} x_{\alpha^{-}}=W_{2}^{k}\left(\mathrm{ReLU}\left(\sum_{\alpha \in C_{\alpha^{-}}}W_{1}^{k}x_{\alpha }\right)\right),\\ \alpha^{-}\in C_{k},k\in \left[1,K\right], \end{array}$$

where $W_{1}^{k},W_{2}^{k}\in R^{d\times d}$ are learnable weights in the *k*-th layer, and $C_{\alpha^{-}}$ denotes the child nodes of $\alpha^{-}$.

**Depth-wise Reparametrization.** In light of the introduction of reparametrization in previous prefix tuning studies [9,11], we propose a depth-wise reparametrization to construct prefix embeddings. First, we take the average of node embeddings at each layer in T as the initial prefix *Q*, formally,

(8) $$Q=\{q_{k}=W_{q}\frac{1}{|C_{k}|}\sum_{\alpha \in C_{k}}x_{\alpha }|k\in \left[1,K\right]\},$$

where $W_{q}\in R^{d\times d}$ denotes a group of learnable parameters. Next, given *B* LM layers, the final prefix $Q=\{q_{k}\cdot \sigma \left(\rho \left(k,b\right)\right)|k\in \left[1,K\right],b\in \left[1,B\right]\}$, where σ(·) denotes the sigmoid function. ρ(k,b) is the relative distance of *k* and *b*, formally,

(9) $$\rho \left(k,b\right)=-\tan[\pi (|\frac{k}{K}-\frac{b}{B}|-0.5)\left(1-\epsilon \right)],$$

where $\epsilon =1e^{-5}$ for avoiding calculation error. The intuition of Equation (9) is that the node embeddings at lower levels of coding tree T would be more suitable for prompting layers next to the input layer in the LM while node embeddings at higher levels, preserving denser information, might be better prefix for layers beneath the output layer.

### 3.3. Model Training and Prediction

**Hierarchy-constrained Prediction.** At the output layer, the LM could predict the logits S that $x_{P}$ corresponds to each label, formally,

(10) $$\begin{array}{c} S=\left\{S^{l}|l\in \left[1,L\right]\right\},S^{l}=\{s_{i}^{l}\left|i\in [1,\right|Y^{l}|]\}, \end{array}$$

where $s_{i}^{l}\in R$ is a single logit. When $s_{i}^{l}>0$, the model will predict the corresponding label $y_{i}^{l}$ as a result. At the inference stage, when the *l*-th layer prompt is observed, we further restrict the model to predict only the labels from $Y^{l}$.

**ZLPR Loss.** A majority of previous methods [1,2,4,14] adopt binary cross entropy (BCE) loss as the optimization target, which intrinsically degrades HTC into a naive multi-label classification. Following recent works [5,6] in HTC, we alternatively utilize the “Zero-bounded Log-sum-exp & Pairwise Rank-based” (ZLPR) loss proposed by [15], formally,

(11) $$L_{\mathrm{ZLPR}}^{l}=\log\left(1+\sum_{i\in \Omega_{\mathrm{pos}}^{l}}e^{-s_{i}^{l}}\right)+\log\left(1+\sum_{j\in \Omega_{\mathrm{neg}}^{l}}e^{s_{j}^{l}}\right),$$

where $\Omega_{\mathrm{pos}}^{l},\Omega_{\mathrm{neg}}^{l}$ denote the target labels and non-target labels in $Y^{l}$, dynamically obtained using the ground truths. ZLPR loss explicitly captures the label correlations in the label hierarchy, which is proved beneficial for HTC [5,6].

**Masked LM Training.** We keep the original masking strategy and loss as BERT [16] to train our model, where 15% words of the text are randomly masked. The final loss $L_{total}$ is the summation of ZLPR loss with regard to classification subtasks $Y^{l}$ at different levels and the MLM loss $L_{MLM}$, formally,

(12) $$L_{total}=\sum_{l=1}^{L}L_{ZLPR}^{l}+L_{MLM}.$$

## 4. Experimental Evaluation

**Datasets and Evaluation Metrics.** Experiments are conducted on four popular datasets in HTC: WOS [17], RCV1-v2 [18], NYTimes [19], and BGC (https://www.inf.uni-hamburg.de/en/inst/ab/lt/resources/data/blurb-genre-collection.html, accessed on 7 May 2024). We randomly split WOS and RCV1-v2 following [6] while keeping the official splits for NYTimes and BGC. The statistics of these datasets are shown in Table 1. Model performance is measured by Micro-F1 and Macro-F1 [20]. Micro-F1 is the harmonic mean of the overall precision and recall of all the test instances, while Macro-F1 is the average F1 score of each category. Thus, Micro-F1 reflects the performance on more frequent labels, while Macro-F1 treats labels equally.

**Implementation Details.** We adopt BERT [16] as the LM to be prompted and update its parameters with Adam [21] as the initial learning rate is $3\times 10^{-5}$. For simplicity, the hidden size *d* of all the learnable weights is set to 768 if not mentioned. The model is trained on a single Nvidia V100 32GB GPU with batch size of 24. Training will be terminated if the Macro-F1 does not increase on the dev set for five epochs. The optimal height *K* of coding trees are [3,2,2,3] for WOS, RCV1-v2, NYTimes, and BGC, respectively. For all text samples, we truncate them to a maximum length of 500.

**Baselines.** All baselines adopt BERT [16]. The performance gaps compared with the vanilla BERT show the significance of their original parts. HiAGM [1], HTCInfoMax [3], HiMatch [2], and HiTIN [14] are all supervised models based on the dual-encoder framework within various techniques to enhance the combination. HGCLR [4] is the pioneer investigation of contrastive learning for HTC while HJCL [5] proposed an instance-wise and label-wise joint supervised contrastive loss. HPT [6] is the inaugural work to introduce prompt tuning for HTC, where a dynamic virtual template and label words are proposed to bridge the gap between MLM and HTC. HBGL [7] pretrains a set of label embeddings with a pretext task and feeds those of ground truth labels to LM during training, which could be treated as a pretrained prompt tuning [22] manner.

### 4.1. Results and Analysis

The main results are shown in Table 2. We can observe that SPIRIT brings 2.26 and 4.69 average improvements to vanilla BERT on Micro-F1 and Macro-F1, respectively. Compared to all the baselines, SPIRIT obtains a state-of-the-art performance on seven out of eight of the metrics. The Macro-F1 performance of SPIRIT is second only to that of HJCL; however, it is notable that HJCL introduces both ZLPR loss and the supervised contrastive learning loss, intrinsically utilizing more supervision signals. In contrast, MLM loss is naturally at a disadvantage due to the self-supervised feature.

Among those prompt tuning methods, SPIRIT outperforms all others across all datasets. Our model significantly outperforms when using datasets with more complex label hierarchies (i.e., RCV1-v2 and NYTimes), while the gaps between it and the runner-up are smaller on WOS. A probable reason is that WOS has only two levels of hierarchy and the ground truths are all single-path, which could not fully present the advantages of structural prefix tuning. Moreover, the ups and downs of HBGL’s ranking on the three datasets prove that its pretrained label embeddings are derived more from semantic information than structural information.

### 4.2. Ablation Studies

**Prefix Tuning Only Works in the Framework of SPIRIT.** To track the source of performance improvements, we conducted ablation studies on both WOS and NYTimes in Table 3. Among the four datasets, WOS has the shallowest label hierarchy (2), while that of NYTimes is the deepest (8). We first replace the structural prefix network (Section 3.2) with a two-layer MLP:$W_{2}\{tanh\left[\left(\left(W_{1}\cdot T\right)\right]\right\}$ to generate the final prefix, where $T=\{t_{1},t_{2},\ldots ,t_{L}\}$ are layerwise prompts (Section 3.1). $W_{1}\in R^{d^{\prime }\times d}$ and $W_{2}\in R^{\left(d\cdot B\right)\times d^{\prime }}$. This is a common prefix tuning network adopted in [9,11]. The performance gaps demonstrate the superiority of structural entropy guided prefixes. Next, we skip Equation (9) and directly feed *B* copies of *Q* to the LM (r.p. Q→B×Q). The degraded results show the necessity of simulating information densification. Further, we replace Q with a random initialized matrix $\xi \in R^{K\times d\times B}$, providing a lower bound as random prefix tuning. The above results jointly suggest the integrity of SPIRIT.

**Figure 3:**
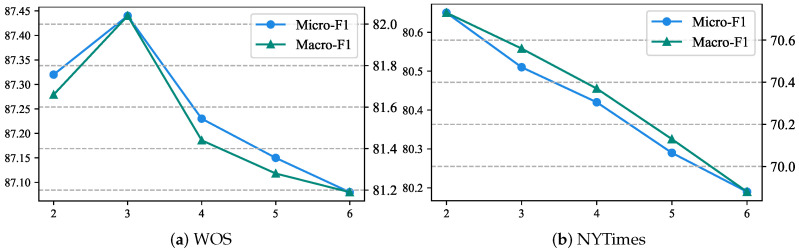
Test performance of SPIRIT with K∈{2,3,4,5,6} on WOS and NYTimes.

**Effects of the *K*-dimensional Structural Entropy.** The only hyperparameter of SPIRIT to be tuned is *K*, which represents the dimension of structural entropy and the height of coding trees. To investigate the effects of *K*, we train SPIRIT with the value of *K* ranges in {2,3,4,5,6} while keeping other settings the same. Figure 3 shows the test performance of different height coding trees on WOS and NYTimes. As *K* grows, the performance of SPIRIT shows a trend of degradation. Higher coding trees may involve more explosive gradients, resulting in unstable optimization.

### 4.3. Evaluation on Path Consistency

**Figure 4:**
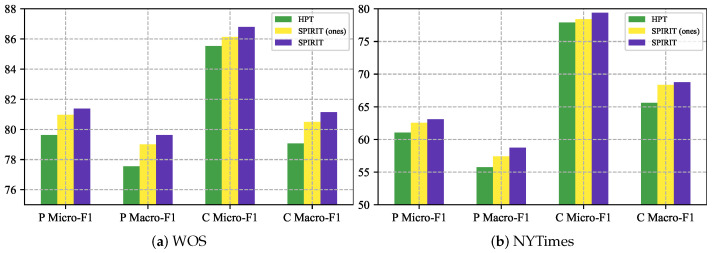
Evaluation on P metrics and C metrics.

We hold the same view as [8,23] that path consistency is crucial to HTC. We further evaluate models with P metrics [8] and C metrics [23], both of which are stricter than the vanilla Micro-F1 and Macro-F1 metrics. In P metrics, only if all labels in one path are predicted accurately is the prediction is considered correct in the confusion matrix. C metrics emphasize that a node label is considered predicted correctly only when all its ancestor nodes are correct. As depicted in Figure 4, our model can better convey the labeling hierarchy at different depths and outperforms HPT in P metrics and C metrics across the board. In comparison with the ablation model “r.p. Q→B×Q”, denoted as “SPIRIT (ones)” in Figure 4, SPIRIT also exhibits superior performance, highlighting the necessity of depth-wise reparameterization (Section 3.2).

### 4.4. Comparison with Large Language Models

Table 4 presents the performance of two emerging large language models, *GPT-3.5-Turbo* [24] and *GPT-4o-0513* [25]. Following [5], we adopt the same prompt template to evaluate the in context learning ability of GPT-4o API (https://openai.com/api/). We also repeat the experiments utilizing a new prompt template presented in Table 5. It can be found that the instruction-tuned large language models are still inferior to the supervised fine-tuned models. Further, we could tell that the reasoning capability of large language models may not generalize to structural information, and the introduction of a structural encoder is necessary.

### 4.5. Qualitative Analysis

**Figure 5:**
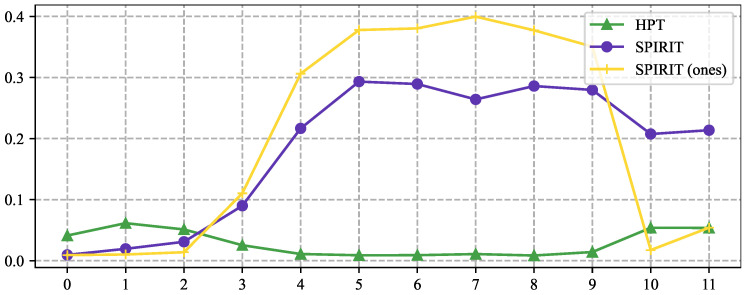
LM attention scores from the text tokens to prompt to the prompt contents.

We illustrate a case study in Figure 5 and Table 6. The test case is from NYTimes [19]. Benefiting from the structural prefix tuning and depth-wise reparameterization, our model exhibits a more profound understanding of the hierarchy, successfully predicting all three paths of ground truths. In contrast, due to providing prompts in the input layer only, HPT rapidly forgets the label hierarchy, resulting in only one predicted path. The attention scores in SPIRIT (ones), surpass SPIRIT at the 3→9-th layer but drop dramatically at layer 10→11. Equally weighted prompts confuse the model output, resulting in only two successfully predicted paths.

**Figure 6:**
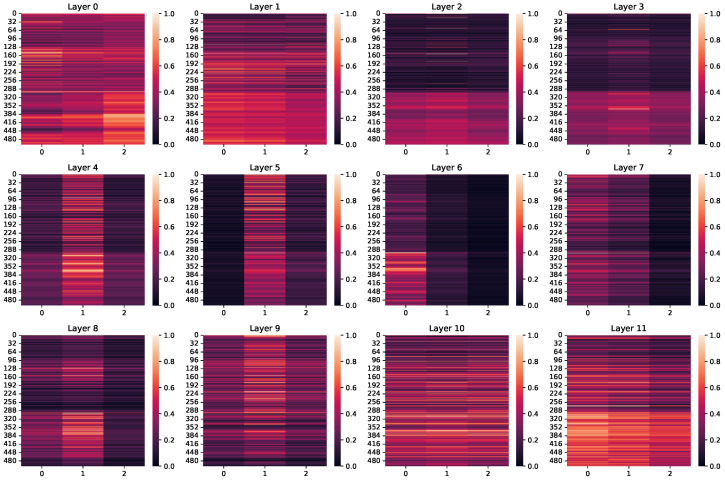
A heat map on attention scores to the prefixes in LM layers. Case: WOS # 619, Ground Truth: [‘Medical’, ‘Menopause’]. Model Prediction: [‘Medical’, ‘Menopause’].

To dynamically demonstrate the effects of structural prefixes and depth-wise reparameterization, we plot the attention scores of text tokens with respect to the structural as heat maps in Figure 6. Structural prefixes are given more attention by the model at both the input and output layers, which demonstrates structural prefix tuning plays an important role in input and output. In the middle layer of the model, influenced by depth-wise reparameterization, the model ignores the zeroth and second prefixes and pays more attention to the intermediate prefixes, which is in line with our expectations.

## 5. Related Work

**Hierarchical Text Classification.** Existing works for HTC could be categorized into local and global approaches [1]. Local approaches build multiple models for labels in different levels in the hierarchy, conveying the information from models in the upper levels to those in the bottom [17,26,27,28]. On the contrary, global studies treat HTC as a flat multi-label classification problem [1,2,3,20,29,30,31,32]. With the advent of pretrained language models (PLMs), numerous studies attempt to solve HTC tasks with PLMs. Techniques derived from pretrained language modeling such as contrastive learning [4,5] and prompt tuning [6,7] are successfully applied to HTC. The boundary between local and global methods is blurring as lots of efforts are paid to utilize the mixture of local and global information [5,7].

**Prompt Tuning.** There are several widely studied prompt tuning manners: (1) Naive prompt tuning, where continuous prompts are directly fed into the input layer for jointly modeling with the original inputs [10,33,34,35]. (2) Prefix tuning, where continuous prompts are not only added to the input but also to the hidden states [9,11]. (3) P-tuning, where a prompt encoder is involved to capture the correlation among continuous prompts [11,36]. (4) Pretrained prompt tuning, where continuous prompts are pretrained together with the language model [22]. Prompt tuning methods for HTC tend to produce continuous prompts based on the label hierarchy. According to the above taxonomy, HPT [6] could be exactly recognized as a p-tuning method since it generates virtual prompt tokens with a graph attention network [13], which acts as the prompt encoder. HBGL [7] could be referred to as a pretrained prompt tuning method where the label embeddings are pretrained under a pretext task along with BERT [16].

**Structural Entropy.** Structural entropy [12] is a natural extension of Shannon entropy [37] in a structural system, capable of measuring the system’s structural complexity. Non-Euclidean data, especially graph data, create a typical structured system. The structural entropy of a graph is defined as the average length of the codewords obtained by a random walk under a specific coding scheme, namely coding tree [12]. In the past few years, structural entropy has been successfully applied in various domains [38,39,40,41,42,43,44,45].

## 6. Conclusions

We introduce SPIRIT, a prefix tuning framework consisting of a structural entropy minimization algorithm, a structural prefix network, and a depth-wise reparameterization strategy. For HTC, structural entropy-guided prefixes could significantly improve the memory of language models for label hierarchy. Depth-wise reparameterization effectively adapts the prefixes to LM layers at different depths. Moreover, random prefixes and repetitive prefixes demonstrate that structural entropy-guided prefix tuning is feasible for improving HTC performance. 

## Figures and Tables

**Table 1:** Dataset statistics. |Y| is the number of classes on the entire label hierarchy. Avg(|Y|) is the number of ground-truth labels per sample. ‘#’ denotes the number of cases in the Train/Dev/Test set.

Dataset	|Y|	Avg(|Y|)	Depth	# Train	# Dev	# Test
WOS	141	2	2	30,070	7518	9397
RCV1-v2	103	3.24	4	20,833	2316	781,265
NYTimes	166	7.60	8	23,345	5834	7292
BGC	146	3.01	4	58,715	14,785	18,394

**Table 2:** Test performance on four datasets. The best results are marked in **bold** while the runner-ups are marked with underline. †: results reported by [4,5]. “-” means not reported or not applicable.

Models	WOS		RCV1-v2		NYTimes		BGC	
	Micro-F1	Macro-F1	Micro-F1	Macro-F1	Micro-F1	Macro-F1	Micro-F1	Macro-F1
Supervised Fine-tuned Methods								
BERT [16] †	85.63	79.07	85.65	67.02	78.24	65.62	78.84	61.19
HiAGM(BERT) [1] †	86.04	80.19	85.58	67.93	78.64	66.76	79.48	62.84
HTCInfoMax(BERT) [3] †	86.30	79.97	85.53	67.09	78.75	67.31	79.16	62.94
HiMatch(BERT) [2] †	86.70	81.06	86.33	68.66	-	-	78.89	63.19
HiTIN(BERT) [14]	87.19	81.51	86.71	69.95	79.65	69.31	-	-
Supervised Contrastive Learning Methods								
HGCLR [4]	87.11	81.20	86.49	68.31	78.86	67.96	79.22	64.04
HJCL [5]	-	-	87.04	**70.49**	80.52	70.02	81.30	66.77
Supervised Prompt Tuning Methods								
HPT [6]	86.94	81.46	87.21	69.32	80.35	70.28	80.27	67.80
HBGL [7]	87.36	82.00	87.23	70.17	80.47	70.19	-	-
**SPIRIT (Ours)**	**87.44**	**82.04**	**87.42**	70.29	**80.65**	**70.73**	**81.88**	**68.60**

**Table 3:** Performance when replacing or removing some components of SPIRIT on the test set of WOS and NYTimes. r.p. denotes the replacement and r.m. denotes remove.

Ablation Models	WOS		NYTimes	
	**Micro-F1**	**Macro-F1**	**Micro-F1**	**Macro-F1**
SPIRIT	87.44	82.04	80.65	70.73
r.m. SPN (Section 3.2)	87.19	81.84	80.27	70.43
r.p. Q→B×Q	87.04	81.47	80.40	70.35
r.p. Q→ξ	86.72	81.34	80.07	70.09
r.m. $L_{MLM}$	86.94	81.56	80.24	70.42
r.p. $L_{ZLPR}\to L_{BCE}$	86.59	80.47	79.45	68.28

**Table 4:** †: results reported by [4,5]. *: Due to resource limitation, we tested the performance of GPTs on randomly sampled 10K cases in RCV1.

Models	WOS		RCV1-v2		NYTimes		BGC	
	**Micro-F1**	**Macro-F1**	**Micro-F1**	**Macro-F1**	**Micro-F1**	**Macro-F1**	**Micro-F1**	**Macro-F1**
GPT-3.5-Turbo [24]	50.30	26.06	45.43 *	26.72 *	37.34	20.41	52.91	34.94
+Prompt in Table 5	48.44	24.98	42.16 *	23.67 *	34.78	18.83	50.06	31.55
GPT-4o-0513 [25]	62.29	51.06	55.91 *	36.47 *	41.55	26.60	63.44	43.03
+Prompt in Table 5	63.17	49.68	59.24 *	33.40 *	43.58	24.78	65.06	42.20
BERT [16] †	85.63	79.07	85.65	67.02	78.24	65.62	78.84	61.19
SPIRIT (Ours)	**87.44**	**82.04**	**87.42**	**70.29**	**80.65**	**70.73**	**81.88**	**68.60**

**Table 5:** We rewrite the prompt in [5].

Prompt Template	You will be given a list of categories formatted as:
	A -> B -> C … -> X
	where A, B, C, X are categories
	and ’->’ represents the parent-child relationship.
	A -> B means A is a child of B.
	B -> C means B is a child of C.
	You should also predict A when you predict B.
	You should also predict B when you predict C.
	[Label Paths]
	Classify the following text into the categories above.
	Just predict the categories, no explanation is allowed.
	Texts: [Input]

**Table 6:** Complete label set for the case study diagram shown in Figure 5.

Methods	Labels
Ground Truths	Art and Design / Classifieds / Job Market / Job Categories Art / Features / Travel / Guides /Activities and Interests Features / Reviews / Art and Design / Arts
HPT	Art and Design / Classifieds / Job Market / Job Categories
SPIRIT (ones)	Art and Design / Classifieds / Job Market / Job Categories Features / Reviews / Art and Design / Arts
SPIRIT	Art and Design / Classifieds / Job Market / Job Categories Art / Features / Travel / Guides /Activities and Interests Features / Reviews / Art and Design / Arts

**Figure A1:**
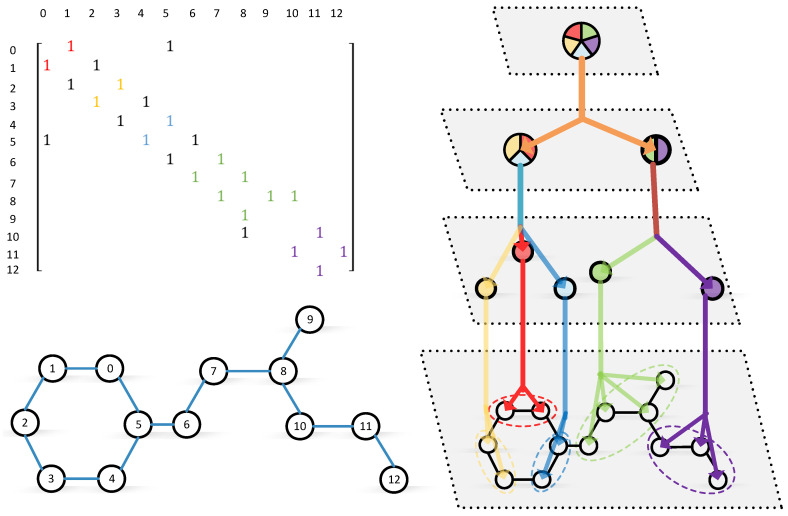
An example of coding tree with height K=3 for synthesized simple graph.

## Data Availability

No new data were created or analyzed in this study.

## Abbreviations

The following abbreviations are used in this manuscript:

HTC	Hierarchical Text Classification
NLP	Natural Language Processing
GNN	Graph Neural Network
MLP	Multi-Layer Perception
LM	Language Model

## Appendix A. More Details

### Appendix A.1. A Notation List for Structural Entropy

To further clarify the definition of structural entropy and coding trees, we provide a notation list in Table A1.

**Table A1:** Notations of Structural Entropy and Coding Trees.

Notation	Explanation
G=(V,E)	A graph *G* with node set *V* and edge set *E*.
T	A coding tree.
α	A non-root node on the coding tree.
$T_{\alpha }$	A subset of *V*, the composition of which depends on α.
$g_{\alpha }$	The number of edges from $T_{\alpha }$ to $V-T_{\alpha }$.
vol(α)	The volume of $T_{\alpha }$, the total degree of nodes in $T_{\alpha }$.
$\alpha^{-}$	The parent node of α on the coding tree T.
$\alpha^{<i>}$	The *i*-th child node of α on the coding tree T.
λ	The root node of coding tree T
$H^{T}\left(G\right)$	The structural entropy of *G*.
$H_{K}\left(G\right)$	The *K*-dimensional structural entropy of *G* when the maximum height of T is *K*.
$C_{k}=\left\{C_{1},\ldots ,C_{m}\right\}$	A partition of *V* in the *k*-th layer of coding tree T.

### Appendix A.2. A Case Study of Algorithm 1

As illustrated in Figure A1, Algorithm 1 generates a coding tree with height K=3 carrying the minimal three-dimensional structural entropy for the 12-node simple graph. The coding tree provides hierarchical partitions of the nodes, revealing the essential structure of the simple graph. The essential structure is abstractive since new nodes are created for partitioning.
